# The effect of adding intrathecal magnesium sulphate to bupivacaine-fentanyl spinal anesthesia: A meta-analysis of randomized controlled trials

**DOI:** 10.1097/MD.0000000000022524

**Published:** 2020-10-02

**Authors:** Jinguo Wang, Zaitang Wang, Bo Shi, Na Wang

**Affiliations:** a Department of Urology, the First Hospital of Jilin University, Changchun, Jilin; b Department of Taxation, School of Public Economics and Administration of Shanghai University of Finance and Economics, Shanghai; c Department of Anesthesiology, the First Hospital of Jilin University Changchun, Jilin, China.

**Keywords:** bupivacaine, fentanyl, magnesium sulphate, meta-analysis, spinal anesthesia

## Abstract

**Trial design:**

The current study is a meta-analysis designed to assess the effect of adding magnesium to a combination of intrathecal bupivacaine and fentanyl.

**Methods:**

The protocol was registered in PROSPERO with the number CRD42020177618. PubMed, Cochrane library, Web of Science, and Google Scholar were searched for randomized controlled trials investigating the effect of adding magnesium to a combination of intrathecal bupivacaine and fentanyl. The continuous data were presented as Ratio of means (RoM). Risk ratio (RR) along with 95% confidence interval (CI) was utilized to assess the dichotomous data.

**Results:**

Ten trials were involved in the present study with 720 adult patients. Compared with control, intrathecal magnesium prolonged time to the first analgesic requirement by an estimate of 1.23 (RoM: 1.23; 95%CI: 1.13–1.33; *P* < .00001), prolonged adequate sensory block duration for surgery by an estimate of 1.16 (RoM: 1.16; 95%CI: 1.05–1.27; *P* = .003), delayed time to maximum sensory level by an estimate of 1.38 (RoM: 1.38; 95%CI: 1.07–1.78; *P* = .01) and reduced the incidence of shivering following spinal anesthesia (risk ratio: 0.38; 95%CI: 0.18 to 0.81, *P* = .01) without influence on time to full motor recovery or incidences of hypotention, bradycardia, nausea, and vomiting or pruritis.

**Conclusion:**

Intrathecal magnesium, when added to a combination of intrathecal bupivacaine and fentany, prolongs the analgesic duration of spinal anesthesia without increased incidences of side effects.

## 1 Introduction

Effective treatment of perioperative pain is important because it can blunt stress reaction, and then lead to a decreased perioperative morbidity.^[1]^ Research continues on techniques and medicines that could provide optimal operative conditions and postoperative pain relief. Various medicines such as opiates, benzodiazepines, the N-methyl D-aspartate (NMDA) receptor antagonists, α_2_ agonists etc, have been used clinically as adjuvants in spinal anesthesia.

The use of small dose of opioid combined with nonopioid drug as adjuvant to local anesthetic in spinal anesthesia is becoming increasingly popular for perioperative pain management. Surgical stimuli can activate NMDA receptors, which are involved in central sensitization.[^2^,^3^] Magnesium, a kind of NMDA receptor antagonist, can block NMDA channels in a voltage-dependent way, and the addition of magnesium can reduce NMDA-induced currents.^[4]^ Therefore, magnesium has antinociceptive effect and has application in spinal anesthesia.

There are an increasing number of papers suggesting that intrathecal magnesium added to bupivacaine-fentanyl spinal anesthesia can improve the anesthetic effect. However, the relative data are inconsistent. Therefore, this meta-analysis is conducted to investigate the effect of adding magnesium to a combination of intrathecal bupivacaine and fentanyl.

## 2 Methods

### 2.1 Search strategy

Neither ethical approval nor informed consent was necessary, since it was a systematic review and meta-analysis. The present study was conducted following the Preferred Reporting Items for Systematic Reviews and Meta-Analyses (PRISMA) recommendations.^[5]^ The protocol was registered in PROSPERO with the number CRD42020177618. Randomized controlled trials (RCTs) investigating the effect of adding magnesium to a combination of intrathecal bupivacaine and fentanyl were selected and reviewed.

### 2.2 Study selection

The literature search was performed by two reviewers in PubMed, Web of Science, Cochrane library, and Google Scholar independently.

The literature search was performed by using the MESH and keywords including: “magnesium”, “fentanyl”, “anesthesia, spinal”, “injection, spinal” and “injection, subarachnoid” without language limitation. We manually searched the reference lists of related papers to find additional eligible RCTs. The latest search was done on March 20, 2020.

RCTs investigating the efficacy of adding magnesium to a combination of intrathecal bupivacaine and fentanyl were sought. The literature research was limited to human studies of subjects aged equal to or more than 18 years. Meeting papers, correspondences or editorials were excluded. If an agreement could not be reached between these 2 reviewers, the opinion of a third reviewer was obtained.

### 2.3 Quality and risk of bias assessment

The risk of bias and the quality of RCTs were separately evaluated using the Cochrane Collaboration Risk of Bias tool and a 5 point Jadad scale by 2 of the reviewers.[^6^,^7^] A score less than 3 was taken as low methodological quality. The third reviewer was consulted when an agreement could not be reached.

### 2.4 Data extraction

Data collection was performed by 2 authors. If an agreement could not be achieved, a third reviewer joined to make a decision. Extracted data included authors, publication year, surgery setting, sample size, dosages of bupivacaine, and fentanyl for spinal anesthesia, magnesium dose, as well as data on block characteristics.

### 2.5 Statistical analysis

Review Manager 5.3 (Cochrane Library, Oxford, England) was utilized for statistical analysis. Because of significant clinical heterogeneity of doses of bupivacaine, fentanyl and magnesium, ratio of means (RoM), standard error, and 95% confidence intervals (CIs) were calculated for continuous data to assess change from baseline for continuous data, under the assumption of equal variances in log scale and log-normal distribution.[^8^,^9^,^10^,^11^] Dichotomous data were analyzed using risk ratio (RR) and CIs. Statistical significance was considered if *P* value was < .05.

## 3 Results

### 3.1 Literature search

**Figure 1:**
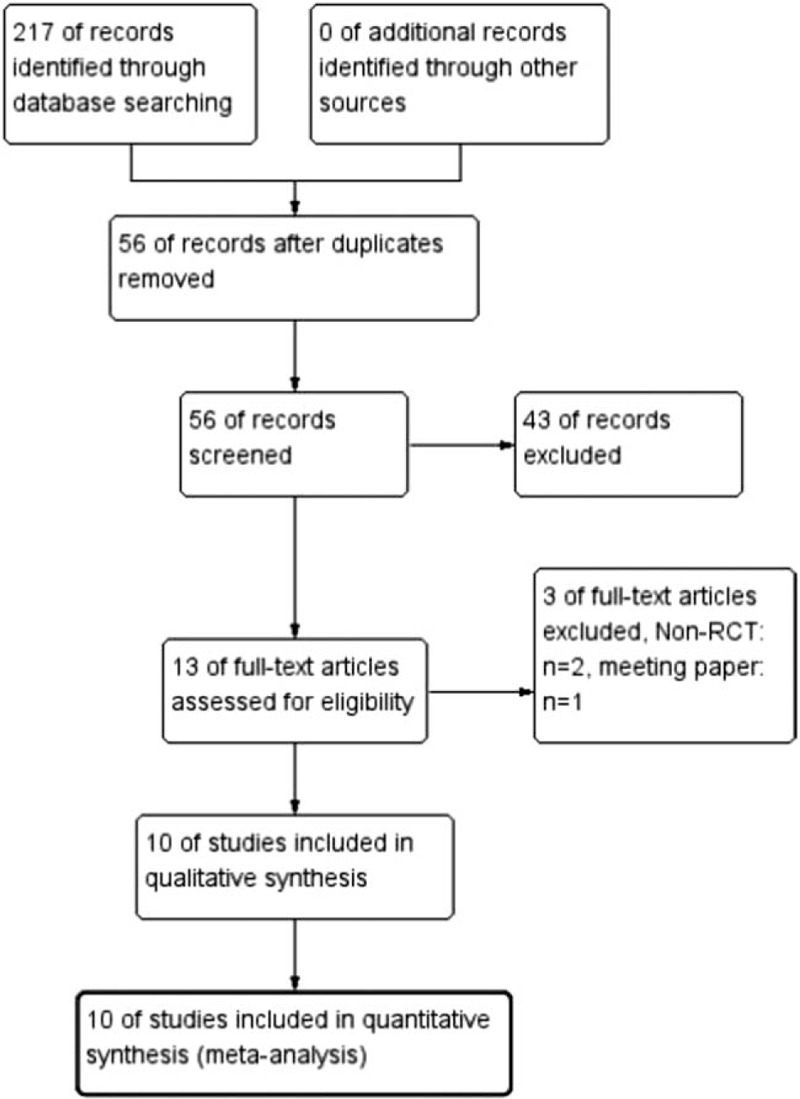
The flow chart of study selection.

Of 165 initial papers found, 149 papers were excluded after screening. Sixteen full-text articles were found and assessed in detail, then 10 RCTs including 720 adult patients were eligible in this meta-analysis.[^12^,^13^,^14^,^15^,^16^,^17^,^18^,^19^,^20^,^21^] The detailed flowchart of the selection was presented in Figure 1.

### 3.2 Study characteristics

**Figure 2:**
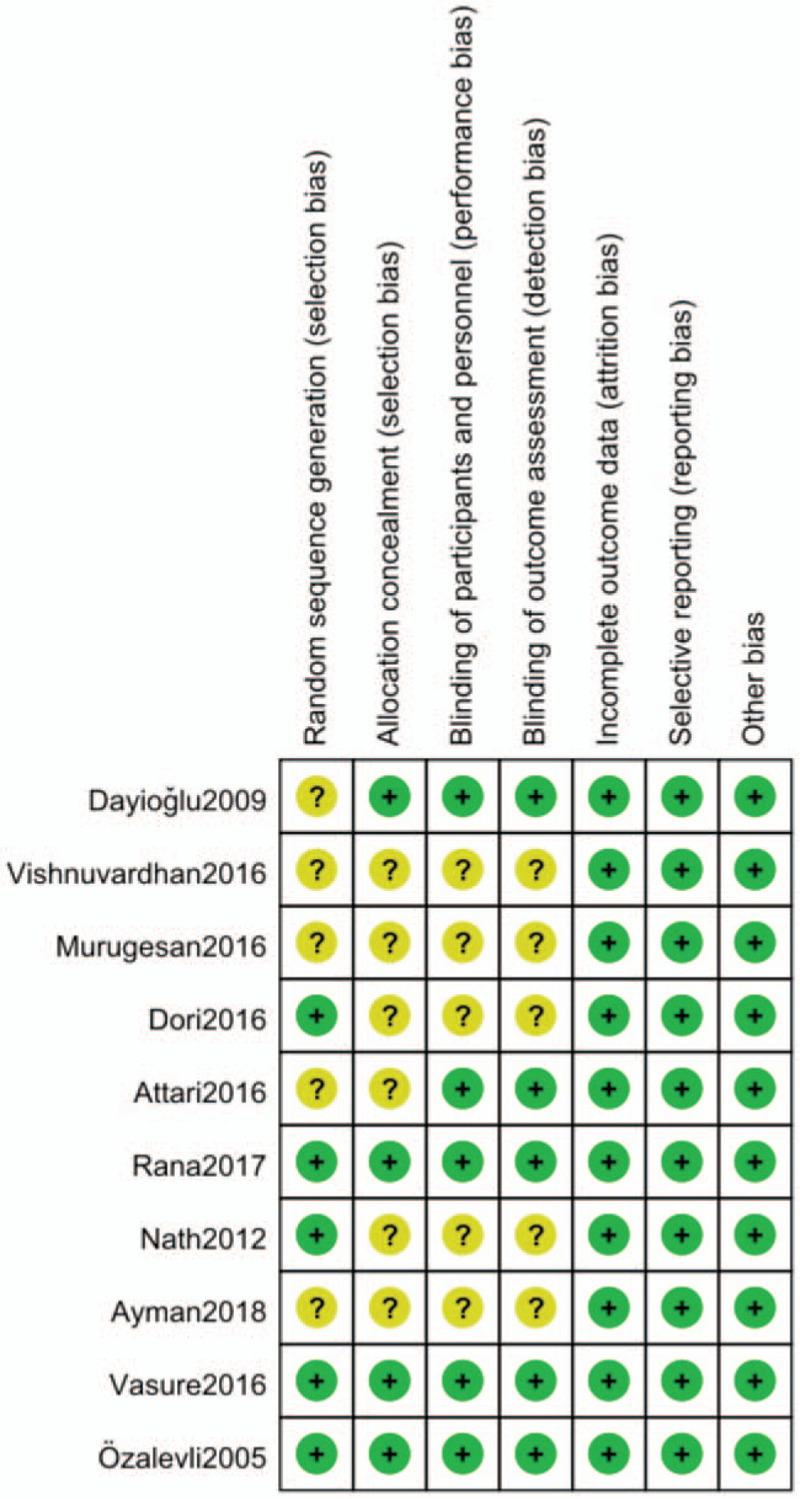
The risk of bias assessment of the included studies.

The details of the eligible RCTs were shown in Table 1. Intrathecal bupivacaine was used in all included trials, and the range of bupivacaine dosages used was 6 to 15 mg. The dosages of fentanyl combined with bupivacaine ranged from 10 to 25 μg. With the exception of one study^[12]^ that used a 100 mg dose of magnesium sulphate, 50 mg magnesium was used in each of the reviewed trials.[^13^,^14^,^15^,^16^,^17^,^18^,^19^,^20^,^21^] The risk-of-bias plot was detailed in Figure 2.

**Table 1:**
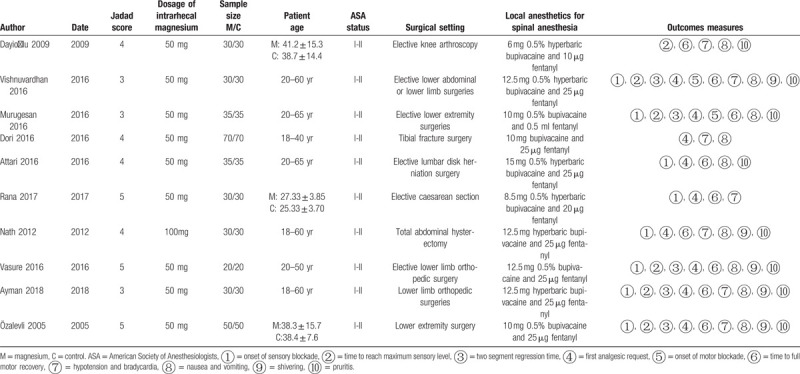
Characteristics of the included randomized controlled trials.

### 3.3 Time to the first analgesic requirement

**Figure 3:**
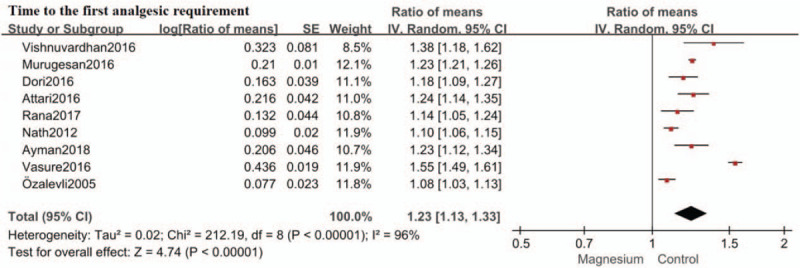
Forest plot for time to the first analgesic requirement. Confidence interval indicates confidence interval; IV = inverse variance, SE = standard error.

The primary outcome was time to the first analgesic requirement which was considered as time period from intrathecal injection to the first analgesic request. Nine studies reported time to the first analgesic requirement.[^12^,^13^,^14^,^15^,^16^,^17^,^18^,^19^,^20^] Intrathecal magnesium prolonged time to the first analgesic requirement by an estimate of 1.23 (RoM: 1.23; 95%CI: 1.13–1.33; *P* < .00001; I^2^ = 96%) compared with control. (Fig. 3) Sensitivity analysis was conducted by removing each study individually. The reliability of the results was confirmed and no source of heterogeneity was found.

### 3.4 Time to maximum sensory level

**Figure 4:**
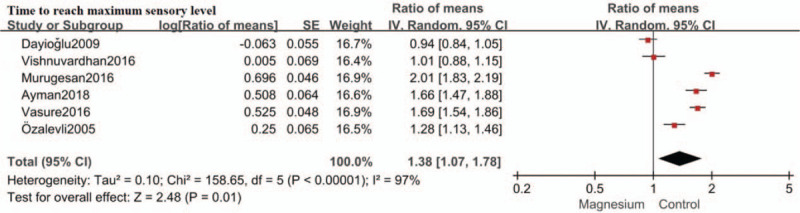
Forest plot for time to reach maximum sensory level. Confidence interval indicates confidence interval, IV = inverse variance, SE = standard error.

Six studies evaluated time to maximum sensory level.[^13^,^14^,^15^,^16^,^17^,^21^] Intrathecal magnesium delayed time to maximum sensory level by an estimate of 1.38 (RoM: 1.38; 95%CI: 1.07–1.78; *P* = .01; I^2^ = 97%) compared with control (Fig. 4). Sensitivity analysis was conducted by removing each study individually. The reliability of the results was confirmed and no source of heterogeneity was found.

### 3.5 Adequate sensory block duration

**Figure 5:**
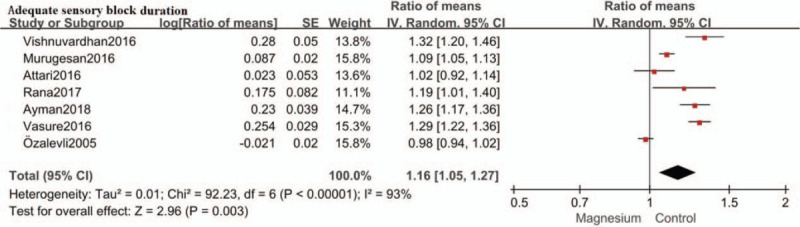
Forest plot for adequate sensory block duration. Confidence interval indicates confidence interval, IV = inverse variance, SE = standard error.

Adequate sensory block duration for surgery was defined as two segment regression time in 5 RCTs[^13^,^14^,^15^,^16^,^17^] and defined as time to T10 regression in 2 RCTs.[^18^,^19^] Therefore, adequate sensory block duration for surgery was assessed in 7 trials.[^13^,^14^,^15^,^16^,^17^,^18^,^19^] Intrathecal magnesium prolonged adequate sensory block duration by an estimate of 1.16 (RoM: 1.16; 95%CI: 1.05–1.27; *P* = .003, I^2^ = 93%) compared with control. (Fig. 5) Sensitivity analysis was conducted by removing each study individually. The reliability of the results was confirmed and no source of heterogeneity was found.

### 3.6 Time to full motor recovery

**Figure 6:**
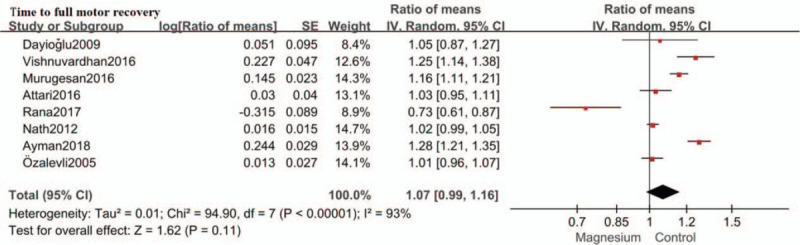
Forest plot for time to full motor recovery. Confidence interval indicates confidence interval, IV = inverse variance, SE = standard error.

The effect of intrathecal magnesium on time to full motor recovery was described in 8 studies reviewed.− No significant difference was found in time to full motor recovery between the magnesium group and the control group (RoM: 1.07; 95%CI: 0.99–1.16; *P* = .11, I^2^ = 93%). (Fig. 6) Sensitivity analysis was conducted by removing each study individually. The reliability of the results was confirmed and no source of heterogeneity was found.

### 3.7 The incidence of hypotention and bradycardia

**Figure 7:**
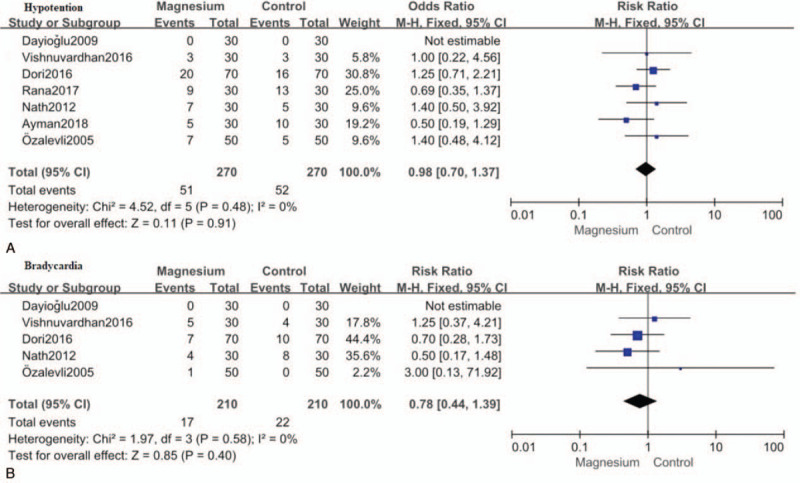
Forest plot for the incidence of hypotention (A) and bradycardia (B). CI = confidence interval, M-H = Mantel-Haenszel.

Seven studies[^12^,^13^,^15^,^17^,^19^,^20^,^21^] reported the incidence of hypotension and 5 studies[^12^,^13^,^17^,^20^,^21^] reported the incidence of bradycardia. For I^2^ = 0%, the fixed effect model was used for meta-analysis. Our study demonstrated that intrathecal magnesium did not increase the incidence of hypotention (RR: 0.98; 95%CI: 0.70–1.37, *P* = .91; I^2^ = 0%) (Fig. 7A) and bradycardia (RR: 0.78; 95%CI: 0.44 to 1.39, *P* = .40; I^2^ = 0%; Fig. 7B), compared with control.

### 3.8 The incidence of nausea and vomiting

**Figure 8:**
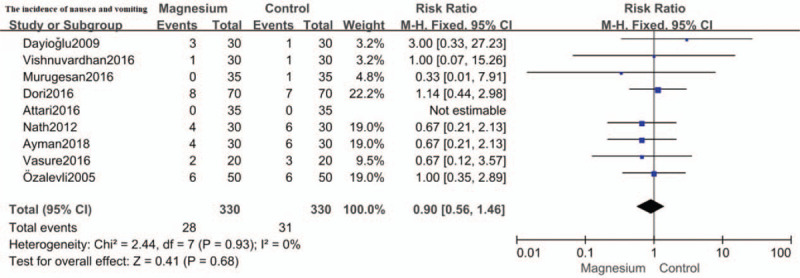
Forest plot for the incidence of nausea and vomiting. CI = confidence interval, M-H = Mantel-Haenszel.

The incidence of nausea and vomiting was reported in all but 1 study.^[19]^ The result showed the difference was not statistically significant in the incidence of nausea and vomiting between the magnesium group and the control group (RR: 0.90; 95%CI: 0.56 to 1.46, *P* = .68; I^2^ = 0%). (Fig. 8)

### 3.9 The incidence of shivering

**Figure 9:**
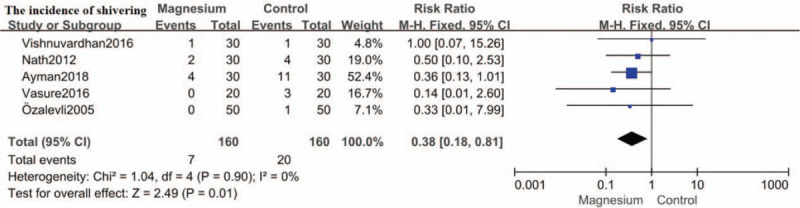
Forest plot for the incidence of shivering. CI = confidence interval, M-H = Mantel-Haenszel.

The incidence of shivering following spinal anesthesia was assessed in 5 trials,[^12^,^13^,^15^,^16^,^17^] permitting quantitative analysis. Intrathecal magnesium was associated with lower incidence of shivering following spinal anesthesia (RR: 0.38; 95%CI: 0.18–0.81, *P* = .01; I^2^ = 0%). (Fig. 9)

### 3.10 The incidence of pruritis

**Figure 10:**
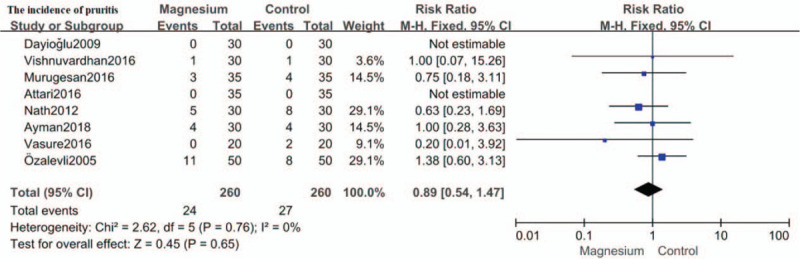
Forest plot for the incidence of pruritis. CI = confidence interval, M-H = Mantel-Haenszel.

The incidence of pruritis was assessed in 8 trials.[^12^,^13^,^14^,^15^,^16^,^17^,^18^,^21^] Compared to placebo, no significant association of intrathecal magnesium with pruritis was found (RR: 0.89; 95%CI: 0.54 to 1.47, *P* = .65; I^2^ = 0%). (Fig. 10)

## 4 Discussion

This meta-analysis demonstrates that addition of intrathecal magnesium is valuable for patients under bupivacaine-fentanyl spinal anesthesia.

The results of this meta-analysis indicate that intrathecal magnesium, when added to a combination of intrathecal bupivacaine and fentany, prolongs time to the first analgesic requirement and adequate sensory block duration for surgery, leads to a significant delay in time to maximum sensory level, and reduces the incidence of post-spinal anesthesia shivering. In addition, intrathecal magnesium sulfate does not influence time to full motor recovery or increase the incidences of hypotension, bradycardia, nausea, and vomiting or pruritis.

Magnesium sulfate is a kind of NMDA receptor antagonist. This prolongation of sensory block resulting from intrathecal magnesium is due to synergistic interaction between intrathecal local anesthetics and NMDA antagonists. Magnesium sulfate can be used as an adjuvant for intrathecal block, because it can diminish neuronal excitation caused by activation of C-fibres.^[22]^ There are evidences suggesting that activation of the NMDA receptors is involved in both hyperalgesia after tissue injury and the development of central sensitization.^[23]^ NMDA receptor antagonists can not only inhibit central sensitization caused by peripheral pain stimulation, but also blunt such hypersensitivity if it is formed up.^[24]^

The safety of intrathecal magnesium has been assessed in rat and canine studies, and no neurological deficit or histopathological change is observed after intrathecal magnesium administration.^[25]^ In this meta-analysis, there are no serious complications associated with intrathecal magnesium reported in the included 10 RCTs.[^12^,^13^,^14^,^15^,^16^,^17^,^18^,^19^,^20^,^21^] Therefore, magnesium seems to be safe for intrathecal administration.

This meta-analysis has 2 limitations. First, statistical heterogeneity is high for some outcomes, making combination of the RCTs debatable, because of various outcome measures among the eligible RCTs. Second, we use the RoM method with assumption of equal variances and lognormal distributions. The assumption is acceptable but can not be absolutely confirmed as lacking of individual patient's data.^[10]^

## 5 Conclusion

Intrathecal magnesium, when added to a combination of intrathecal bupivacaine and fentany, prolongs the analgesic duration of spinal anesthesia, without increased incidences of side effects.

## Author contributions

XXX.
